# Hemodynamic effects of cycle ergometry versus dynamic unilateral arm exercise during right heart catheterization: a prospective observational study

**DOI:** 10.1186/s12890-026-04848-1

**Published:** 2026-11-14

**Authors:** Eugen Berezucki, Maria Heinrich, Alexander Rueß, Tobias J. Lange, Markus Perl, Stefan Stadler

**Affiliations:** 1 Department of Internal Medicine II, University Medical Center Regensburg, Franz-Josef-Strauß-Allee 11, 93053 Regensburg, Germany; 2 Department of Internal Medicine II, District Hospital Bad Reichenhall, Bad Reichenhall, Germany; 3 Department of Internal Medicine III, University Medical Center Regensburg, Regensburg, Germany

**Keywords:** Dynamic arm exercise, right heart catheter, Pulmonary circulation, Pulmonary hypertension, Hemodynamic effects

## Abstract

**Background:**

The reintroduction of exercise pulmonary hypertension (EPH) to the pulmonary hypertension (PH) definition by the European guidelines underlines the importance of examining the hemodynamic response to exercise during right heart catheterization. Because the standard exercise modality of cycle ergometry (CE) is not universally accessible, we compared its hemodynamic effects with those of dynamic single-arm exercise (AE) in patients undergoing right heart catheterization (RHC).

**Methods:**

50 consecutive patients with indication for RHC were enrolled. Hemodynamic parameters were assessed at rest, followed by randomized exercise periods with an intervening rest phase. Thermodilution was used to determine cardiac output. The intensity of the AE was patient-determined, whereas CE was conducted at a fixed work rate ranging from 10 to 50 watts, based on the patient’s physical status. Oxygen uptake (VO₂) was estimated using the reverse Fick principle to compare relative exercise intensity between modalities. EPH was defined by an mPAP/cardiac output slope > 3 mmHg·L⁻¹·min⁻¹, and exercise-induced left heart disease by a rise in PAWP from ≤ 15 mmHg at rest to > 25 mmHg during exercise.

**Results:**

Mean pulmonary arterial pressure increased by 12 mmHg with CE compared to 3 mmHg with AE (p<0.001), pulmonary artery wedge pressure increased by 5 mmHg versus 2 mmHg (p<0.001), cardiac output increased by 2.2 L/min versus 0.6 L/min (p<0.001) and mean pulmonary vascular resistance decreased by 0.05 WU with CE (p=0.761) and by 0.32 with AE (p=0.03). Estimated VO₂ during AE reached only 24% of the increase observed during CE, corresponding to an external workload equivalent of approximately 6 W. No patient showed an increase in pulmonary artery wedge pressure during exercise above 25mmHg to unmask left heart disease. In patients without PH at rest, CE led to the diagnosis of EPH in 5 patients while the used AE protocol was not suitable to detect EPH.

**Conclusion:**

AE proved to be a safe exercise modality in our study but, with the protocol applied here, produced significantly smaller hemodynamic changes during RHC than CE and is thus not an adequate substitute in this form, leaving open whether an intensity-matched protocol could achieve comparable hemodynamic effects.

## Background

Pulmonary hypertension (PH) is defined by an elevated mean pulmonary artery pressure (mPAP) > 20 mmHg, potentially resulting in right heart failure.

Since in the early stages of PH the symptoms occur only during physical stress, additional information might be obtained with exercise testing during right heart catheter (RHC). Not only is exercise testing a useful method to unmask left heart disease, but it is also mandatory to diagnose exercise pulmonary hypertension (EPH). Furthermore, exercise testing also carries information about the patient’s prognosis [1–3].

The most common form of physical stress during a right heart catheterization is cycle ergometry (CE). However, CE is not available in many catheterization laboratories or is not adequately possible due to femoral venous access for RHC or orthopedic difficulties, therefore other forms of exercise modalities are necessary [4–6].

Some studies compared static handgrip exercise as well as isotonic dumbbell exercise as an exercise testing modality with a primary goal to unmask latent left heart disease with CE during RHC [7, 8]. Handgrip produced significantly smaller hemodynamic changes and is not suitable as a substitute for CE [9].

However, CE is a dynamic exercise modality, characterized by rhythmic contractions and relaxations. The cardiovascular response to dynamic exercise is markedly different to static exercise, characterized by sustained contraction of muscles without change in muscle length (e.g. handgrip), therefore we chose a similar exercise, but for the upper body [10].

An approach that is both cost-effective and space-efficient is dynamic arm loading using a dumbbell. While holding a 1-kg dumbbell, the patient performs concentric and eccentric arm movements. The intensity of this dynamic arm exercise (AE) is determined by the patient, with the objective of maintaining continuous movement for a duration of 5 min. AE during RHC has been classified as safe by previous studies [5, 7, 11], however none of these studies investigated the effects of AE during RHC in patients with known or suspected PH.

The aim of the present study was to investigate whether dynamic single arm contractions is an alternative stress testing modality to CE, so it can be used to unmask latent left heart disease or diagnose EPH.

## Materials and methods

### Patients and study design

In this prospective single-center study patients with an indication for RHC due to suspected PH were enrolled.

Procedures were conducted between July 2023 and April 2025 in the RHC laboratory of the University Medical Center Regensburg, a national PH referral center. Hemodynamics were measured at rest and during two exercise modalities in randomized order, separated by a resting phase. Randomization was performed using Microsoft Excel version 2208. Patients unable to complete one modality were excluded from analysis.

Pulmonary hypertension was defined as mean pulmonary arterial pressure (mPAP) > 20 mmHg at rest. Exercise-induced left heart disease was defined as an increase in pulmonary arterial wedge pressure (PAWP) from ≤ 15 mmHg at rest to > 25 mmHg during exercise [12].

EPH is defined by an abnormal rise in mPAP relative to cardiac output (mPAP/CO slope > 3 mmHg·min·L⁻¹).

Patients of this study only participated after informed consent. The study was in line with the declaration of Helsinki and was approved by the local ethics committee.

### Exercise modalities

For the AE, a 1‑kg dumbbell was utilized. Prior to initiation of the RHC, the patient was instructed in the correct execution of a rhythmic concentric and eccentric movement and had to demonstrate proper form. The patient was expected to perform both exercises continuously for a minimum of five minutes. CE was conducted at a constant workload of 10 to 50 watts, adjusted according to the patient’s estimated exercise capacity based on patient’s history. Patients were instructed to maintain a cadence of approximately 60 revolutions per minute throughout CE.

### Right heart catheterization

RHC was performed using a balloon-tipped, flow-directed catheter (Swan-Ganz, Edwards Lifesciences, Irvine, CA, USA), which was inserted via the antecubital or internal jugular vein and advanced under fluoroscopic guidance when required. Patients were positioned semi-recumbent, and the cycle ergometer was adjusted to individual body height. The pressure transducer was leveled at the mid-thoracic position and calibrated to atmospheric pressure. Hemodynamic pressures were recorded as digitally derived mean values. Cardiac output (CO) was determined by thermodilution, averaging a minimum of three measurements with less than 10% variability. Pulmonary vascular resistance (PVR) was calculated as (mPAP – PAWP)/CO. Continuous monitoring included electrocardiography, pulse oximetry, and non-invasive measurement of blood pressure.

Following catheter placement and a resting period of at least five minutes, baseline measurements were obtained, including systolic (sPAP), diastolic (dPAP), and mPAP (digitally derived), PAWP, right atrial pressure (RAP), cardiac output, non-invasive systolic (SBP), diastolic (DBP), and mean arterial pressure (MAP), heart rate (HR), peripheral oxygen saturation (SpO2) and mixed venous oxygen saturation (SvO2) as well as lactate concentration, the latter two assessed by blood gas analysis. Cardiac Index (CI) was calculated by dividing the CO by the body surface area.

Subsequently, the first of two exercise protocols was initiated, as determined by randomization. Hemodynamic recordings commenced after at least 180 s of exercise, once invasively measured pressure values had stabilized [13]. Data collection followed a standardized sequence both at rest and during exercise: (1) pulmonary artery pressures and RAP simultaneously, (2) PAWP, (3) CO, (4) HR, (5) SvO2/SpO2, (6) lactate concentration and (7) non-invasive blood pressure. Following completion of the first exercise protocol, patients underwent a resting interval until pulmonary pressures had returned to baseline, which was documented before proceeding. Thereafter, the second exercise protocol was performed with identical measurements.

If indicated, vasoreactivity testing or stepwise oximetry was subsequently conducted according to institutional standards (not reported here). The procedure was completed upon catheter removal.

### Estimation of exercise intensity

To estimate the relative exercise intensity of the two modalities, oxygen uptake (VO₂) was calculated using the reverse Fick principle: VO₂ (ml/min) = CO (L/min) × 13.4 × Hb (g/dL) × (SaO₂ − SvO₂), where SaO₂ and SvO₂ are expressed as fractions (0–1). SpO₂ was used as a surrogate for arterial saturation and Hb was taken from the value measured under each condition.

### Statistical analysis

The study’s data were analyzed using Prism version 10.6.0. To assess significant differences between measurements at rest and during exercise, and between various exercise tests, paired-sample t-tests were used. Additionally linear regression models assessed correlations, while Bland-Altman diagrams evaluated the agreement between mPAP and PAWP values for the two exercise types. All statistical comparisons considered a p-value below 0.05 as significant, and no adjustments for multiple comparisons were made.

## Results

### Patient characteristics

Between July 2023 and April 2025 50 consecutive patients with established or suspected PH were included. Out of those, 24 were female (48%), and their mean age was 67 ± 10 years. Most patients (*n* = 35, 70%) had PH at rest, of which 8 (23%) were assigned to group 1, 6 (17%) to group 2, 8 (23%) to group 3, 8 (23%) to group 4, and 4 (11%) to group 5. One patient with unclassified PH was not assigned to a Nice group. Among all patients with PH 27 (77%) were diagnosed with precapillary PH, 4 (11%) with postcapillary PH, 3 (9%) with combined post- and precapillary PH and 1 (3%) with unclassified PH. The clinical and hemodynamical classification was made according to current guidelines.

Further baseline characteristics are shown in Table 1.

**Table 1:** Patient characteristics

Parameter	All (*n* = 50)
Female sex, n (%)	24 (48)
Age, years	67 ± 10
Height, cm	171 ± 9
Body weight, kg	78 ± 15
Body mass index, kg/m^2^	26.9 ± 4.8
Current/Previous smoker, n (%)	22 (44%)
Arterial hypertension, n (%)	32 (64%)
Hypercholesterolemia, n (%)	26 (52%)
Diabetes mellitus, n (%)	8 (16%)
Interstitial lung disease, n (%)	11 (22%)
Chronic obstructive pulmonary disease (%)	7 (14%)
History of deep vein thrombosis, n (%)	7 (14%)
History of pulmonary embolism, n (%)	11 (22%)
Atrial fibrillation, n (%)	15 (30%)
Coronary heart disease, n (%)	18 (36%)
Echocardiography parameters	
RVEDD1 (mm)	43 ± 10
TAPSE (mm)	21 ± 6
Right atrial surface (cm^2^)	23 ± 10
Vena cava inferior diameter (mm)	17 ± 5
sPAP (mmHg)	48 ± 20
TAPSE/sPAP ratio	0.57 ± 0.37
LVEF (%)	58 ± 6

All metric data given as mean ± standard deviation

RVEDD = right ventricular enddiastolic diameter; TAPSE = tricuspid annular plane systolic excursion; LVEF= left ventricular ejection fraction

### Hemodynamics under different exercise modalities

Exercise testing was performed in 50 patients. The average exercise duration for each type was 7 ± 3 min with an average rest of 6 ± 3 min to recover to the resting mPAP value. During CE, patients exercised at an average load of 25 ± 6 watts and maintained a cadence of around 60 revolutions per minute. The intensity of AE was self-selected, with instructions to choose a cadence sustainable for five minutes while still challenging, as seen in previous studies [6]. No complications occurred during any measurements. However, in 10 patients a valid PAWP value could not be obtained during at least one exercise modality and these patients were excluded from paired t-test analysis of PAWP and PVR.

During CE, all hemodynamic parameters changed markedly from resting values, except for PVR, which remained similar with 3.55 ± 2.45 WU at rest vs. 3.50 ± 2.49 WU during ergometry (*p* = 0.761). mPAP and PAWP rose from 29 ± 13 mmHg to 41 ± 16 mmHg (*p* < 0.001) and from 11 ± 5 mmHg to 16 ± 7 mmHg (*p* < 0.001), respectively. Cardiac output increased from.

5.1 L/min ± 1,5 to 7.3 ± 2.6 L/min (*p* < 0.001) and calculated VO₂ rose from 234 ± 47 ml/min to 623 ± 181 ml/min. Other hemodynamic variables also changed significantly as detailed in Table 2.

**Table 2:** Hemodynamic parameters during rest and exercise

Parameter	Rest	Cycle exercise	Arm-exercise	*p*-value (cycle exercise vs. rest)	*p*-value (arm exercise vs. rest)	*p*-value (cycle exercise vs. arm exercise)
HR, 1/min	69 ± 11 (46–94)	93 ± 18 (59–155)	79 ± 17 (49–136)	< 0.001	< 0.001	< 0.001
MAP, mmHg	97 ± 12 (70–124)	103 ± 13 (68–144)	100 ± 13 (69–128)	< 0.001	0.036	0.019
mPAP, mmHg	29 ± 13 (11–65)	41 ± 16 (17–90)	32 ± 14 (12–70)	< 0.001	< 0.001	< 0.001
PAWP, mmHg	11 ± 5 (2–27)	16 ± 7 (5–39)	13 ± 6 (3–32)	< 0.001	< 0.001	< 0.001
RAP, mmHg	7 ± 3 (2–14)	11 ± 6 (3–32)	9 ± 4 (3–25)	< 0.001	0.008	< 0.001
CO, l/min	5.1 ± 1.5 (2.2–8.9)	7.3 ± 2.6 (3.0-13.9)	5.7 ± 1.7 (2.2–9.7)	< 0.001	0.001	< 0.001
CI, l/min/m^2^	2.6 ± 0.7 (1.2–3.8)	3.8 ± 1.1 (1.7–5.9)	3.0 ± 0.8 (1.2–4.8)	< 0.001	< 0.001	< 0.001
PVR, WU	3.55 ± 2.45 (0.82–10.30)	3.50 ± 2.49 (0.70-10.23)	3.23 ± 2.28 (0.70–8.97)	0.761	0.03	0.170
S_v_O_2_, %	68 ± 7 (49–80)	44 ± 13 (16–76)	62 ± 10 (29–77)	< 0.001	< 0.001	< 0.001
S_p_O_2_, %	95 ± 3 (90–100)	93 ± 5 (80–100)	95 ± 3 (88–100)	< 0.001	0.309	< 0.001
Lactate, mg/dl	6 ± 3 (3–12)	15 ± 9 (3–46)	10 ± 5 (3–30)	< 0.001	< 0.001	< 0.001
Estimated VO₂, ml/min	234 ± 47 (142–336)	623 ± 181(306–1155)	327 ± 68 (176–443)	< 0.001	< 0.001	< 0.001

All metric data given as mean ± standard deviation (min-max)

During AE mPAP and PAWP increased to 32 ± 14 mmHg (*p* < 0.001) and 13 ± 6 mmHg (*p* < 0.001), respectively. The PVR fell from 3.55 ± 2.45 to 3.23 ± 2.28 (*p* = 0.03). SpO₂ did not significantly change during AE (Table 2).

In comparison to exercise testing on a cycle ergometer, hemodynamic changes were less pronounced during AE: Heart rate increased to 93 ± 18 bpm during CE and to 79 ± 17 bpm during AE (*p* < 0.001). The mPAP was 41 ± 16 mmHg during CE and 32 ± 14 mmHg during AE (*p* < 0.001). PAWP rose to 16 ± 7 mmHg with CE and 13 ± 6 mmHg with AE (*p* < 0.001). Peak cardiac output was 7.3 ± 2.6 L/min for CE and 5.7 ± 1.7 L/min for AE (*p* < 0.001). VO₂ rose to 327 ± 68 ml/min with AE and to 623 ± 181 ml/min with CE. Mixed venous oxygen saturation (SvO₂) decreased from 68 ± 7% at rest to 44 ± 13% during CE (*p* < 0.001) and to 62 ± 10% during AE (*p* < 0.001). Concordant with the reduction in SvO2, lactate levels rose to 15 ± 9 mg/dl during CE (*p* < 0.001) but only to 10 ± 5 mg/dl during AE with 5.9 ± 2.5 mg/dl being the value at rest. Only the PVR was not statistically significant different between exercise modalities with 3.50 ± 2.49 WU during CE versus 3.23 ± 2.28 WU during AE (*p* = 0.170) (Table 2).

### Unmasking latent left heart disease

None of the patients with a resting PAWP < 15mmHg reached at least 25mmHg under any of the exercise modalities.

### Exercise pulmonary hypertension

Across the 15 patients without resting PH, the mPAP/CO slope averaged 3.76 ± 2.98 mmHg·min·L⁻¹ during CE (median 2.52), with no negative values, versus 1.99 ± 6.28 (median 0.86; range − 11.77 to 14.29) during AE, including three negative slopes. Applying the > 3 mmHg·min·L⁻¹ threshold, CE flagged 5 patients and AE 4, with one patient flagged by both. In all four AE-flagged patients cardiac output rose by ≤ 0.6 L/min (Table 3).

**Table 3:** Hemodynamic values for patients without PH at rest

Patient	mPAP rest	mPAP CE	mPAP AE	CO rest	CO CE	CO AE	Slope CE	Slope AE
1	14	20	15	6.73	10.27	7.57	1.69	1.19
2	14	24	16	5.00	8.60	7.67	2.78	0.75
3	17	26	18	4.50	6.50	4.90	4.50	2.50
4	15	18	14	2.90	5.63	4.07	1.10	-0.85
5	17	25	17	5.93	9.20	7.37	2.45	0.00
6	16	21	16	5.77	8.60	6.60	1.77	0.00
7	11	17	12	4.53	6.67	4.60	2.80	14.29
8	18	29	18	3.60	6.03	4.20	4.53	0.00
9	19	25	23	5.90	10.00	8.07	1.46	1.84
10	20	35	19	5.50	7.00	5.83	10.00	-3.03
11	14	22	21	7.33	10.50	7.83	2.52	14.00
12	18	28	21	6.80	11.80	7.40	2.00	5.00
13	18	39	20	3.27	5.30	3.67	10.34	5.00
14	12	19	14	4.27	5.33	4.10	6.60	-11.76
15	20	30	21	8.57	13.90	9.73	1.88	0.86
**Mean ± standard deviation**							3.76 ± 2.97	1.99 ± 6.28
**Median**							2.52	0.86
**Range (min–max)**							1.10 to 10.34	-11.76 to 14.29
**Negative slopes**							0	3

mPAP given in mmHg, CO in L/min, slope in mmHg·min·L⁻¹. Slope calculated as (mPAP during exercise − mPAP at rest) / (CO during exercise − CO at rest). CE = cycle ergometry; AE = arm exercise; CO = cardiac output; mPAP = mean pulmonary arterial pressure; PH = pulmonary hypertension

### Relative exercise intensity

Estimated oxygen uptake rose from 234 ± 47 ml/min at rest to 623 ± 181 ml/min during cycle ergometry and 327 ± 68 ml/min during arm exercise. The exercise-induced rise in VO₂ during arm exercise was 24% of that during cycle ergometry (93 ± 46 vs. 390 ± 154 ml/min; *p* < 0.001). Calibrated against the mean ergometer workload of 25 W, arm exercise corresponded to an equivalent external load of approximately 6 W.

**Fig. 1:**
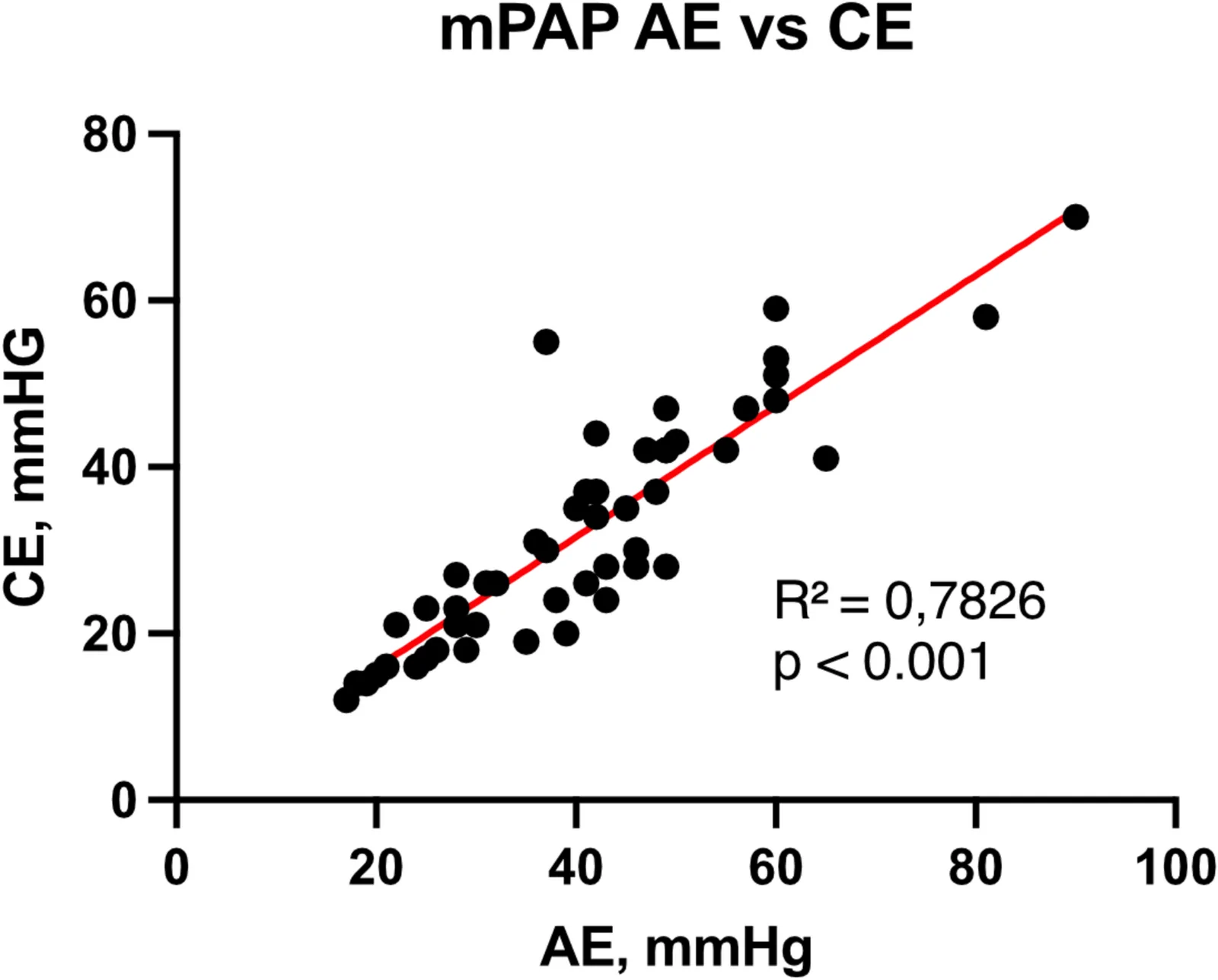
Correlation of mean pulmonary artery pressures between different exercise modalities. This linear regression model depicts the relationship between mPAP values obtained using the various exercise methods, and this relationship is statistically significant (p<0.001)

**Fig. 2:**
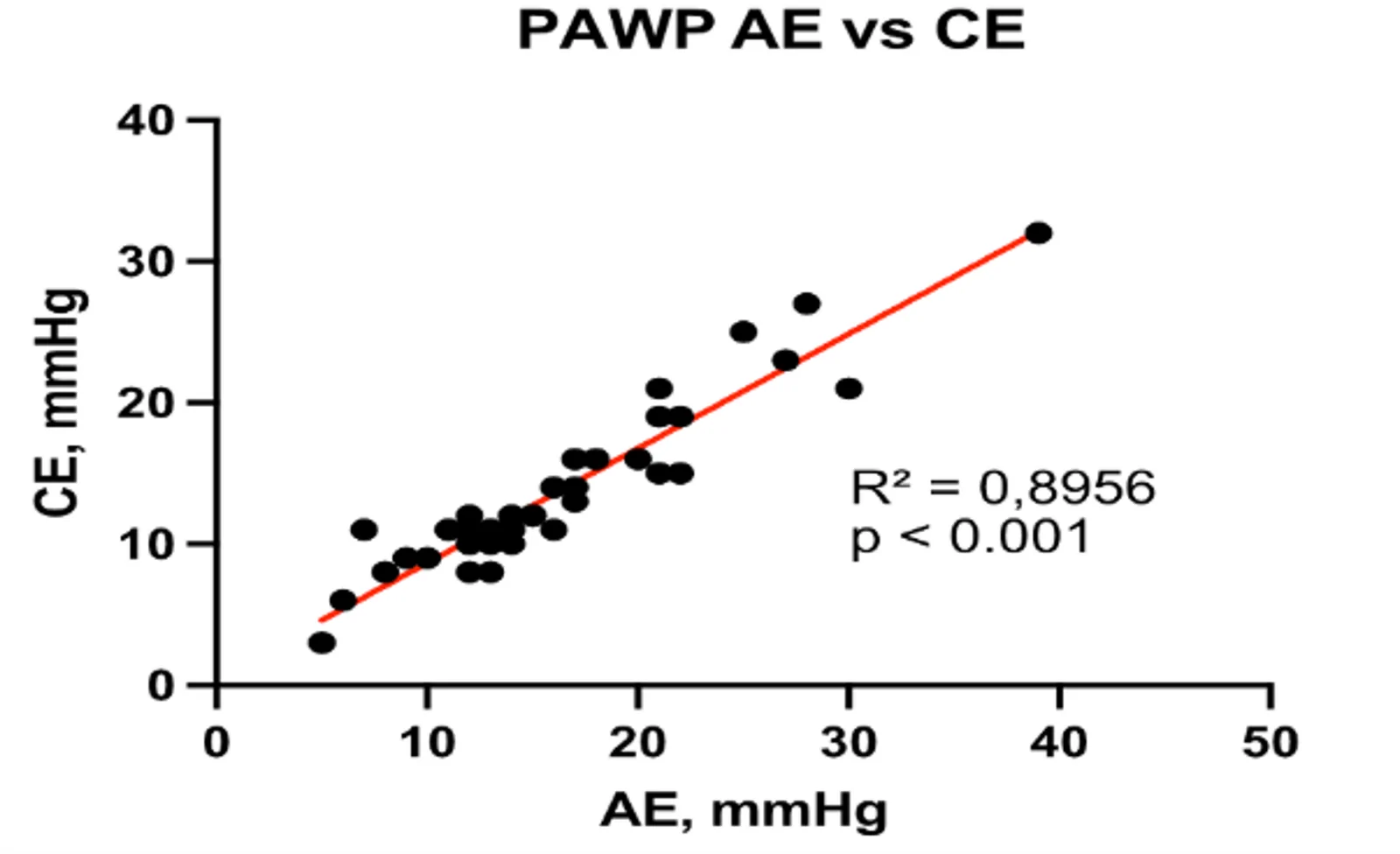
Correlation of pulmonary wedge pressures between different exercise modalities. This linear regression model depicts the relationship between PAWP values obtained using the various exercise methods, and this relationship is statistically significant (p<0.001)

**Fig. 3:**
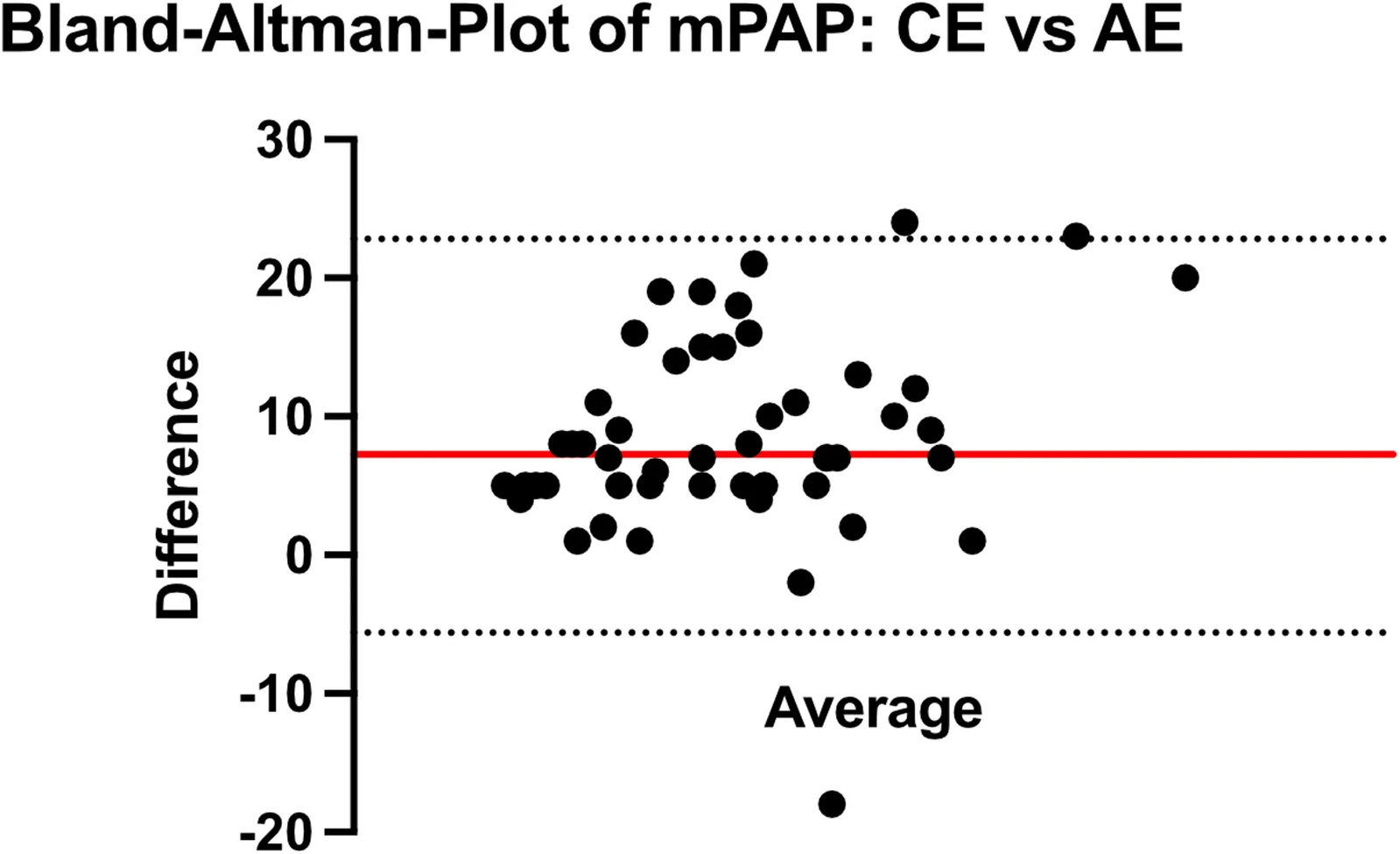
Comparison of mean pulmonary artery pressures between different exercise modalities. This Bland-Altman plot graphs the difference between mPAP measurements taken during CE and AE against their average values (mPAP CE + mPAP AE) / 2). The 95% confidence limits of agreement span from -5.6 mmHg to 22.8 mmHg (mean of 9 mmHg ± 1.96 × 7.3 mmHg). A mean difference of 9 mmHg (CE minus AE) reveals a systematic overestimation of mPAP during CE compared to AE

**Fig. 4:**
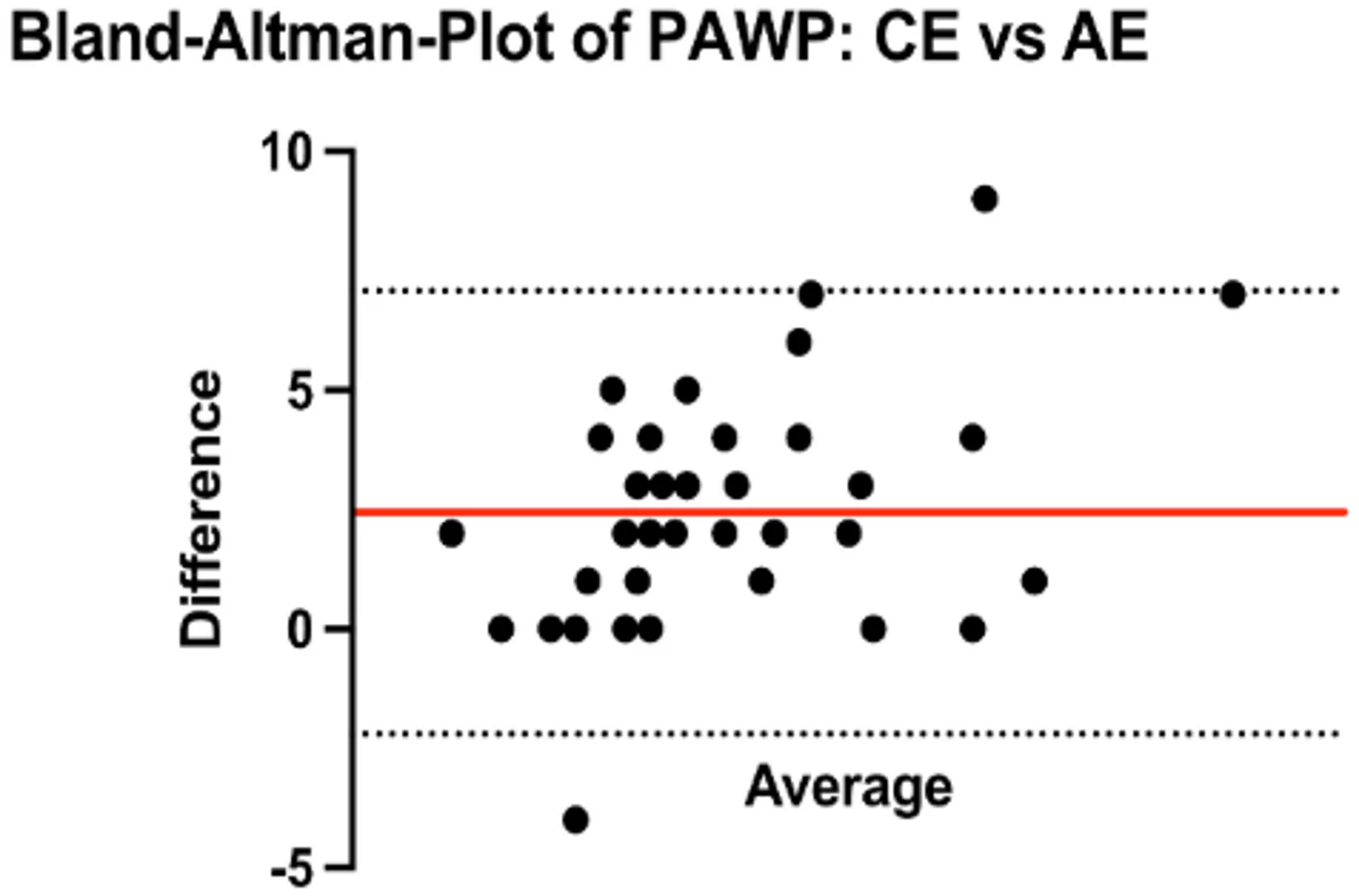
Comparison of pulmonary wedge pressures between different exercise modalities. This Bland-Altman plot graphs the difference between PAWP measurements taken during CE and AE against their average values (PAWP CE + PAWP AE) / 2). The 95% confidence limits of agreement span from -2.2 mmHg to 7.1 mmHg (mean of 2 mmHg ± 1.96 × 2.4 mmHg). A mean difference of 2 mmHg (CE minus AE) reveals a systematic overestimation of PAWP during CE compared to AE

This lower stimulus during arm exercise was reflected in the pressure measurements, which tracked closely between modalities yet were consistently higher under cycle ergometry. mPAP values during CE and AE showed a strong and significant positive correlation (R² = 0.7826, *p* < 0.001; Fig. 1), as did PAWP values (R² = 0.8956, *p* < 0.001; Fig. 2). Bland-Altman analysis revealed a systematic bias with consistently higher values during CE: the mean difference for mPAP was 9 mmHg (limits of agreement − 5.6 to 22.8 mmHg; Fig. 3) and for PAWP 2 mmHg (limits of agreement − 2.2 to 7.1 mmHg; Fig. 4).

## Discussion

The present study included 50 patients with either diagnosed or suspected PH who underwent RHC. Both exercise modalities, CE as well as AE, demonstrated significant changes in mPAP, PAWP, RAP, CO, HR, MAP, S_v_O_2_, estimated VO2 and venous lactate concentrations compared to the resting state, with significantly greater changes with CE. S_p_O_2_ showed only significant changes during CE.

PVR changed little with either modality (3.55 to 3.50 WU with CE and to 3.23 WU with AE); the nominal significance for AE alone (*p* = 0.03) is not supported by the direct comparison between modalities (*p* = 0.17) and most likely represents chance rather than a genuine physiological difference.

Because AE intensity was self-selected while CE was performed at a fixed workload, we used the reverse Fick principle to estimate VO₂ in each condition, allowing the two workloads to be compared directly. The exercise-induced rise in VO₂ during AE reached only 24% of that during CE, corresponding to an estimated 6 watts of AE workload against a mean CE load of 25 watts. This intensity mismatch, rather than a fundamental limitation of dynamic arm exercise as a modality, likely accounts for much of the attenuated hemodynamic response observed with AE.

Linear regression supports this conclusion. mPAP and PAWP correlated strongly between the two modalities (R² = 0.78 and R² = 0.90; Figs. 1 and 2).

However CE values were systematically higher on Bland-Altman analysis, with wide limits of agreement for mPAP (mean difference 9 mmHg, 95% limits − 5.6 to 22.8 mmHg; Fig. 3) and narrower, though still relevant, limits for PAWP (mean difference 2 mmHg, 95% limits − 2.2 to 7.1 mmHg; Fig. 4).

While the systematic bias is consistent with the lower AE intensity, the width of the limits shows that the current protocol is not interchangeable with CE at the individual level. How much of this width stems from the variable, self-selected AE intensity rather than from modality-intrinsic differences cannot be resolved here and should be addressed by a standardized intensity-matched protocol.

These factors are consistent with the poor diagnostic performance of AE for EPH. The mPAP/CO slope is a two-point construct with ΔCO in the denominator. Because AE raised cardiac output by only 0.6 L/min on average, small variation in that denominator produced disproportionate and occasionally negative slopes (range − 11.77 to 14.29 mmHg·min·L⁻¹). All four patients flagged by AE had a cardiac output increase of ≤ 0.6 L/min, as did the single patient flagged by both modalities, whose AE cardiac output rose by only 0.4 L/min; the concordance with CE is therefore most likely coincidental. AE in this form does not yield interpretable slopes for diagnosis of exercise PH.

None of the patients with a normal resting PAWP crossed the 25 mmHg threshold during either exercise modality. This likely reflects that patients were referred for suspected pulmonary hypertension rather than recruited for unexplained dyspnea with intermediate HFpEF probability, together with the individually titrated (not symptom-limited maximal) workloads and the semi-recumbent position — factors that reduce sensitivity for unmasking occult left heart disease compared to dedicated HFpEF-provocation protocols [3, 14].

The hemodynamic changes between the resting state and after CE were similar to other studies [2]. There are no studies which compared the hemodynamic effects of any form of AE during RHC to CE, the most common exercise modality.

Some studies used AE, as no CE was available or not possible, but not with a validated exercise protocol, and never in comparison with CE [4, 6, 7].

However, there are several studies measuring non-invasive cardiorespiratory function comparing upper vs. lower body exercise. A meta-analysis by Larsen et al. compared the oxygen uptake during cycle and arm ergometry and showed a linear association between upper and lower body ergometry with lower values during arm ergometry [15].

Motoji et al. compared the effects of handgrip, AE with a dumbbell and CE on cardiorespiratory parameters and non-invasively measured hemodynamic parameters and showed greater hemodynamic response with cycling than with dumbbell exercise, while handgrip showed the smallest change in parameters [16].

The present study with invasive measurements aligns and reinforces the results of the noninvasive studies.

We acknowledge certain limitations of our study. First, cardiac output was not determined using the direct Fick method, which remains the gold standard for invasive assessment of pulmonary hemodynamics. Second, no fixed cadence was obligatory during AE, allowing participants to select their preferred frequency and therefore intensity. Third, the workload during CE was not identical for all the patients. Although this approach reflects more physiological conditions, it may have resulted in interindividual differences in workload distribution and muscular efficiency. Finally, the patient cohort was heterogeneous, which may limit the generalizability of the findings to more uniform pulmonary hypertension populations.

## Conclusion

Our study demonstrated that dynamic, unilateral arm exercise using a 1 kg dumbbell at a patient-selected cadence is a safe and feasible form of stress during RHC. The hemodynamic changes observed during AE were less pronounced than those seen with CE; therefore, this AE protocol cannot be considered suitable replacement for CE. Whether a standardized upper-body protocol with a matched workload to cycle ergometry produces equivalent hemodynamic changes remains to be tested.

## Data Availability

The data that support the findings of this study are available from the corresponding author upon reasonable request.

## Abbreviations

AE: dynamic single–arm exercise (arm exercise)
APC: article processing charge
CE: cycle ergometry
CI: cardiac index
CO: cardiac output
dPAP: diastolic pulmonary arterial pressure
DBP: diastolic blood pressure
EPH: exercise pulmonary hypertension
ERS: European Respiratory Society
ESC: European Society of Cardiology
Hb: hemoglobin
HFpEF: heart failure with preserved ejection fraction
HR: heart rate
LVEF: left ventricular ejection fraction
MAP: mean arterial pressure
mPAP: mean pulmonary arterial pressure
PAWP: pulmonary arterial wedge pressure
PH: pulmonary hypertension
PVR: pulmonary vascular resistance
RAP: right atrial pressure
RHC: right heart catheterization
RVEDD: right ventricular enddiastolic diameter
SaO₂: arterial oxygen saturation
SBP: systolic blood pressure
sPAP: systolic pulmonary arterial pressure
SpO₂: peripheral oxygen saturation
SvO₂: mixed venous oxygen saturation
TAPSE: tricuspid annular plane systolic excursion
VO₂: oxygen uptake
WU: Wood units

## References

[1] Zeder K, Banfi C, Steinrisser-Allex G, Maron BA, Humbert M, Lewis GD, et al. Diagnostic, prognostic and differential-diagnostic relevance of pulmonary haemodynamic parameters during exercise: a systematic review. Eur Respir J. 2022;60:2103181. 10.1183/13993003.03181-2021. PMC9556812 35332069

[2] Kovacs G, Humbert M, Avian A, Lewis GD, Ulrich S, Vonk Noordegraaf A, et al. Prognostic relevance of Exercise Pulmonary Hypertension: Results of the multi-center PEX-NET Clinical Research Collaboration. Eur Respir J. 2024;2400698. 10.1183/13993003.00698-2024. PMC11684422 39603672

[3] Eisman AS, Shah RV, Dhakal BP, Pappagianopoulos PP, Wooster L, Bailey C, et al. Pulmonary Capillary Wedge Pressure Patterns During Exercise Predict Exercise Capacity and Incident Heart Failure. Circ Heart Fail. 2018;11:e004750. 10.1161/CIRCHEARTFAILURE.117.004750. PMC5937988 29695381

[4] Steen V, Chou M, Shanmugam V, Mathias M, Kuru T, Morrissey R. Exercise-Induced Pulmonary Arterial Hypertension in Patients With Systemic Sclerosis*. Chest. 2008;134:146–51. 10.1378/chest.07-2324. 18403670

[5] Jain CC, Egbe AC, Reddy YNV, Connolly HM, Hagler DJ, Miranda WR. Role of Exercise During Invasive Hemodynamic Assessment in Symptomatic Adults With a Systemic Right Ventricle. JACC Adv. 2022;1:100023. 10.1016/j.jacadv.2022.100023. PMC11198355 38939324

[6] Borlaug BA, Nishimura RA, Sorajja P, Lam CSP, Redfield MM. Exercise hemodynamics enhance diagnosis of early heart failure with preserved ejection fraction. Circ Heart Fail. 2010;3:588–95. 10.1161/CIRCHEARTFAILURE.109.930701. PMC3048586 20543134

[7] King ML, Dracup KA, Fonarow GC, Woo MA. The hemodynamic effects of isotonic exercise using hand-held weights in patients with heart failure. J Heart Lung Transpl Off Publ Int Soc Heart Transpl. 2000;19:1209–18. 10.1016/s1053-2498(00)00208-4. 11124492

[8] Hamatani Y, Iguchi M, Minami K, Ishigami K, Ikeda S, Doi K, et al. Isometric Handgrip Stress Test during Right Heart Catheterization in Patients with Mitral Regurgitation -A Case Series Study. Intern Med Tokyo Jpn. 2022;61:1817–22. 10.2169/internalmedicine.8505-21. PMC9259811 34776492

[9] Rueß A, Stadler S, Stark T, Berezucki E, Lange TJ. Hemodynamic effects of cycle ergometry and low-intensity handgrip in patients with pulmonary hypertension. Front Med. 2026;13:1727173. 10.3389/fmed.2026.1727173. PMC12975984 41822894

[10] Martinez MW, Kim JH, Shah AB, Phelan D, Emery MS, Wasfy MM, et al. Exercise-Induced Cardiovascular Adaptations and Approach to Exercise and Cardiovascular Disease. J Am Coll Cardiol. 2021;78:1453–70. 10.1016/j.jacc.2021.08.003. 34593128

[11] Balady GJ, Jacobs AK, Faxon DP, Ryan TJ. Dynamic arm exercise during cardiac catheterization in the assessment of stenotic valvular disease. Clin Cardiol. 1990;13:632–7. 10.1002/clc.4960130909. 2208822

[12] Humbert M, Kovacs G, Hoeper MM, Badagliacca R, Berger RMF, Brida M, et al. 2022 ESC/ERS Guidelines for the diagnosis and treatment of pulmonary hypertension. Eur Respir J. 2023;61:2200879. 10.1183/13993003.00879-2022. 36028254

[13] Fletcher GF, Ades PA, Kligfield P, Arena R, Balady GJ, Bittner VA, et al. Exercise Standards for Testing and Training: A Scientific Statement From the American Heart Association. Circulation. 2013;128:873–934. 10.1161/CIR.0b013e31829b5b44. 23877260

[14] Fudim M, Kittipibul V, Swavely A, Gray A, Mikitka J, Young E, et al. Discrepancy in the Diagnosis of Heart Failure With Preserved Ejection Fraction Between Supine Versus Upright Exercise Hemodynamic Testing. Circ Heart Fail. 2024;17. 10.1161/CIRCHEARTFAILURE.124.012020. 39513293

[15] Larsen RT, Christensen J, Tang LH, Keller C, Doherty P, Zwisler A-D, A SYSTEMATIC REVIEW AND META-ANALYSIS COMPARING CARDIOPULMONARY EXERCISE TEST VALUES OBTAINED FROM THE ARM CYCLE AND THE LEG CYCLE RESPECTIVELY IN HEALTHY ADULTS, et al. Int J Sports Phys Ther. 2016;11:1006–39. PMC5159627 27999717

[16] Motoji Y, Forton K, Pezzuto B, Faoro V, Naeije R. Resistive or dynamic exercise stress testing of the pulmonary circulation and the right heart. Eur Respir J. 2017;50:1700151. 10.1183/13993003.00151-2017. 28705944

